# Signaling role of iron in NF-kappa B activation in hepatic macrophages

**DOI:** 10.1186/1476-5926-2-S1-S36

**Published:** 2004-01-14

**Authors:** Shigang Xiong, Hongyun She, Hidekazu Tsukamoto

**Affiliations:** 1 Department of Pathology, Keck School of Medicine, University of Southern California, USA; 2 Greater Los Angeles VA Health Care Systems, Los Angeles, California, USA

## Abstract

Iron is both essential and toxic for cells and impaired iron homeostasis has been shown to cause or potentiate various forms of liver injury. Research in our laboratory suggests that iron also plays a pivotal role in intracellular signaling for NF-kappa B activation in hepatic macrophages (HM). Our results showed: 1) HM from alcohol-fed rats had a increase in the nonheme iron content accompanied by NF-kappa B activation; 2) iron chelation normalized nonheme iron concentration and blocked enhanced NF-kappa B activation and TNF-alpha expression in these cells; 3) LPS-induced NF-kappa B activation was also blocked by iron chelator; 4) iron directly induced TNF-alpha expression via IKK and NF-kappa B activation in normal HM. We propose that iron acts as an independent proinflammatory molecule via induction of the intracellular signaling for NF-kappa B activation in HM and primes the liver for chronic inflammation and injury.

## Iron and HM NF-kappa B activation in alcohol model

Our earlier study showed that hepatic macrophages (HM) from rats fed ethanol and high fat diet had a significant 70% increase in the nonheme iron content as compared to controls [1]. This study also suggested enhanced heme turnover as a cause of the increased iron storage in HM. To test this notion, an increase in HM iron content was recapitulated in vitro by phagocytosis of heat-treated autologous red blood cells. To extend this observation to the whole animal situation, the effects of splenectomy on alcohol-fed animals were also examined. The most intriguing and critical finding from these cellular or animal model experimentations, was that activation of NF-kappa B was tightly correlated with the increased non-heme iron content in HM, suggesting the priming role of iron in NF-kappa B activation and proinflammatory cytokine expression by HM in alcoholic liver disease.

## Direct iron induction of TNF-alpha in cultured HM

**Figure 1:**
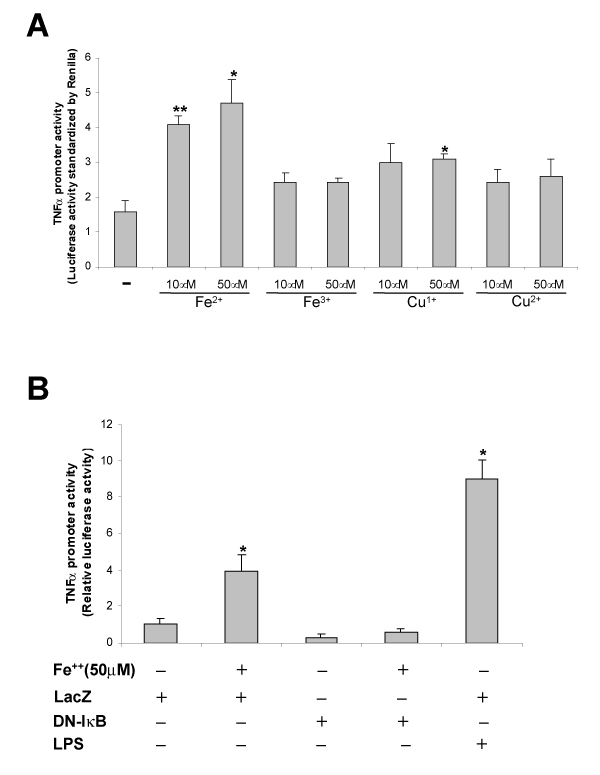
(A.) Cultured HM were transfected with a TNF-alpha promoter-lucigerase construct followed by the treatment with Fe^2+^, Fe^3+^, Cu^1+ ^or Cu^2+^. The data were normalized by co-transfected Renilla luciferase activity. Note the Fe2+ induces the promoter activity by 2–3 fold at 10 and 50 micromolar. Cu^1+ ^slightly induces but oxidized metals (Fe^3+ ^and Cu^2+^) do not. (B.) HM were co-transfected with the promoter-luciferase construct with a vector of super-repressor I-kappa B-alpha (DN-I-kappa B), followed by addition of Fe^2+^. Note DN-I-kappa B completely blocks iron induced promoter activity.

Direct addition of ferrous but not ferric iron in cultured HM increased TNF-alpha release 8 fold at 10 and 50 micromolar during a 4 hr treatment period without cell toxicity. Cuprous (Cu^1+^) but not cupric (Cu^2+^) copper also stimulated TNF-alpha release at 50 micromolar to less extent. Thus, these results demonstrate direct stimulation of HM TNF-alpha release by iron and copper in a redox status dependent manner. We then tested whether Fe^2+ ^stimulates TNF-alpha promoter in cultured HM. The promoter activity was indeed increased 2~3 fold with 10~50 micromolar Fe^2+^. Cu^1+ ^(50 micromolar) also slightly increased TNF-alpha promoter activity but not Cu^2+ ^or Fe^3+ ^(Figure 1). Co-transfection of a super repressor I-kappa B-alpha vector completely abrogated the stimulation with 50 micromolar Fe^2+ ^(Figure 1). The enhanced promoter activity with 50 micromolar Fe^2+ ^was about half of the maximal response achieved with LPS (500 ng/ml) in a serum-free condition. These results establish that Fe^2+ ^activates TNF-alpha promoter in a NF-kappa B dependent manner.

## Iron-induced activation of IKK and NF-kappa B and ROS generation in cultured HM

**Figure 2:**
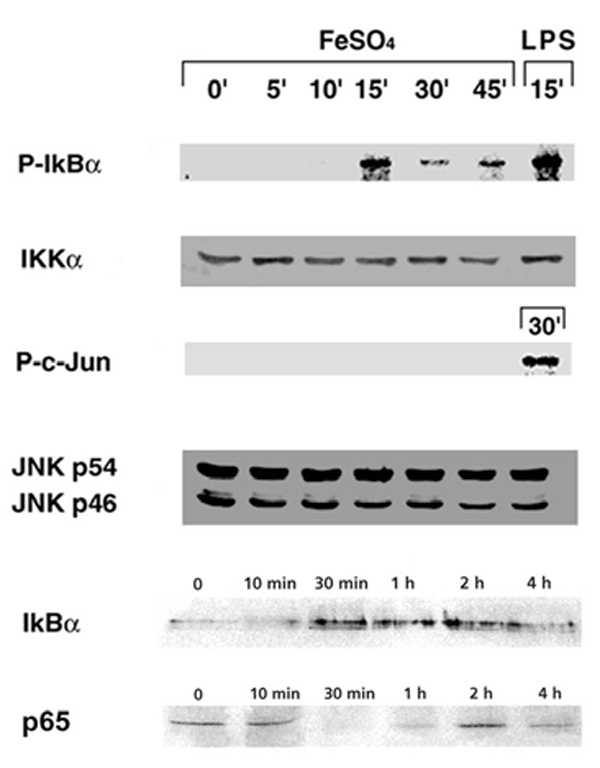
I-kappa B kinase (IKK) and c-Jun NH2-terminal kinase (JNK) activity were determined on HM lysate collected at different time points after FeSO_4 _treatment. Note that IKK activity as assessed by phosphoylation of GST-I-kappa B-alpha (P-I-kappa B-alpha), is increased at 15 min after addition of iron while no activation of JNK is evident.

As shown in the top panel of Figure 2, IKK activity, as assessed by phosphorylation of GST-I-kappa B-alpha, was increased at 15 min after addition of ferrous iron. The timing of IKK activation preceded a disappearance of cytosolic I-kappa B-alpha and an increase in the nuclear level of p65 at 30~45 min. Iron did not induce JNK and p38 activities (unpublished results). These results correlated well with induced NF-kappa B binding in iron-treated HM at 30 min and no effects on AP-1 binding in these cells (data not shown). Thus, these results unequivocally demonstrate that ferrous iron can directly and selectively stimulates the signaling leading to IKK and NF-kappa B activation in cultured HM. Addition of ferrous iron to these cells resulted in an enhancement of the hydroxyl and methyl-POBN adduct signals. Both signals increased with incubation time up to a maximum at 15–20 min, which coincided with IKK activation at 15~30 min and preceded activation of NF-kappa B at 30 min, suggesting the possible signaling role of the former in the latter events. In fact, this notion was made by previous studies which demonstrated activation of NF-kappa B by hydroxyl radical generating systems and a reversal of this effect by hydroxyl radical scavengers or metal chelators in Jurkat cells [2].

## Discussion and Conclusion

Our studies to date strongly suggest the causal link between iron and activation of NF-kappa B in HM in both normal and alcohol-fed rats. These findings raise a question as to how iron signals to activate NF-kappa B. Since NF-kappa B is a redox sensitive transcription factor and ROS are implicated in its activation [3,4], it is reasonable to speculate that iron stimulates ROS production in HM and in turn ROS activates NF-kappa B. Conversely, stimulation of HM with an agonist such as LPS, induces ROS generation and ROS may initiate intracellular signaling that is dependent on a chelatable pool of iron. Nitric oxide (NO) is known to cause mobilization of intracellular iron [5] and to inhibit enzymes with catalytically active iron-sulfur groups [6]. Superoxide anion can also release iron from ferritin [7]. Indeed, our preliminary results demonstrate that a selective inhibitor of iNOS and Cu/Zn SOD overexpression abolish a LPS-mediated transient rise in the intracellular level of chelatable iron and NF-kappa B activation (unpublished observation). We propose that iron acts as a proinflammatory effector molecule via selective induction of the intracellular signaling for NF-kappa B activation and that dysregulation of this signaling mechanism may prime HM for chronic liver inflammation and injury.
